# Emergence and evolution of canonical microRNAs: A case study in *Arabidopsis halleri* and *A. lyrata*

**DOI:** 10.1093/plcell/koaf159

**Published:** 2025-06-16

**Authors:** Pei Qin Ng

**Affiliations:** Assistant Features Editor, The Plant Cell, American Society of Plant Biologists; Department of Plant Sciences, University of Cambridge, Cambridge, CB2 3EA Cambridgeshire, UK

MicroRNAs (miRNAs) are a class of small RNAs that are 21 or 22 nucleotides (nts) long and have been shown to play critical roles in various plant life processes, including growth and immunity (Meyers and Axtell 2019; Lopez-Gomollon and Baulcombe 2022). Canonical miRNAs are generated through a 2-step cleavage process conducted by DICER-LIKE 1 (DCL1) from longer, imperfectly paired, hairpin-like RNA precursors (Axtell et al. 2011; Lopez-Gomollon and Baulcombe 2022; Mencia et al. 2023). The resulting mature miRNA duplex is preferentially loaded onto ARGONAUTE 1 (AGO1) proteins, forming the functional RNA-induced Silencing Complex (RISC). This complex then recognizes and silences target mRNAs through sequence complementarity. Interestingly, the miRNA population is dynamic and continuously evolving.

Unlike canonical miRNAs, which exhibit well-defined characteristics and biogenesis pathways, recently evolved or “young” miRNAs tend to have lower expression levels, less defined processing preferences, and often lack validated mRNA targets (Axtell et al. 2011; Cuperus et al. 2011). Early studies on miRNA evolution focused mainly on protein-coding genes and comparisons between distantly related plant species, limiting our understanding of the evolution of young miRNAs into canonical miRNAs.

In a recent study, Pavan and colleagues (Pavan et al. 2025) conducted small RNA sequencing on *Arabidopsis lyrata* and its closely related species *A. halleri*, which diverged approximately one million years ago (Ramos-Onsins et al. 2004). Given the fragmented state of the existing *A. halleri* genome, the authors generated a new high-quality, chromosome-level assembly for this study. Both the newly assembled *A. halleri* and the latest *A. lyrata* genome were annotated for miRNAs using a combination of ShortStack and MiRkwood pipelines. To validate the functionality of predicted miRNAs AGO1 and AGO4, immunoprecipitation assays were used to confirm loading, a signature feature of biologically active miRNAs. After filtering, 558 and 374 candidate miRNAs were identified in *A. halleri* and *A. lyrata*, respectively, with most miRNAs associated with AGO1.

Pavan et al. also addressed the saturation point for miRNA detection and the required sequencing depth. The authors estimated that 165 million reads are needed to detect 90% of the predicted young miRNAs, while at least 20 million reads are sufficient to detect highly expressed miRNAs. Via random subsampling, they found that miRNA detection in both species had not yet reached saturation due to limited accession numbers. These insights are valuable for guiding experimental design in studies of miRNA and other small RNAs.

The authors identified miRNAs conserved across distant plant species, within the Brassicaceae family, and among *A. halleri*, *A. lyrata*, and their common ancestor, *A. thaliana*. They found shared miRNAs between *A. helleri* and *A. lyrata*, as well as species-specific ones. For example, 58% of *A. halleri* miRNAs (*n* = 207) and 40% of *A. lyrata* miRNAs (*n* = 91) were species specific. These represent evolutionarily young miRNAs, given the short divergence time between species. Further characterization using linear regression modeling showed that young miRNAs tend to have lower expression, longer hairpin precursors, greater structural stability, and lower processing precision compared to canonical miRNAs (Figure).

**Figure.**
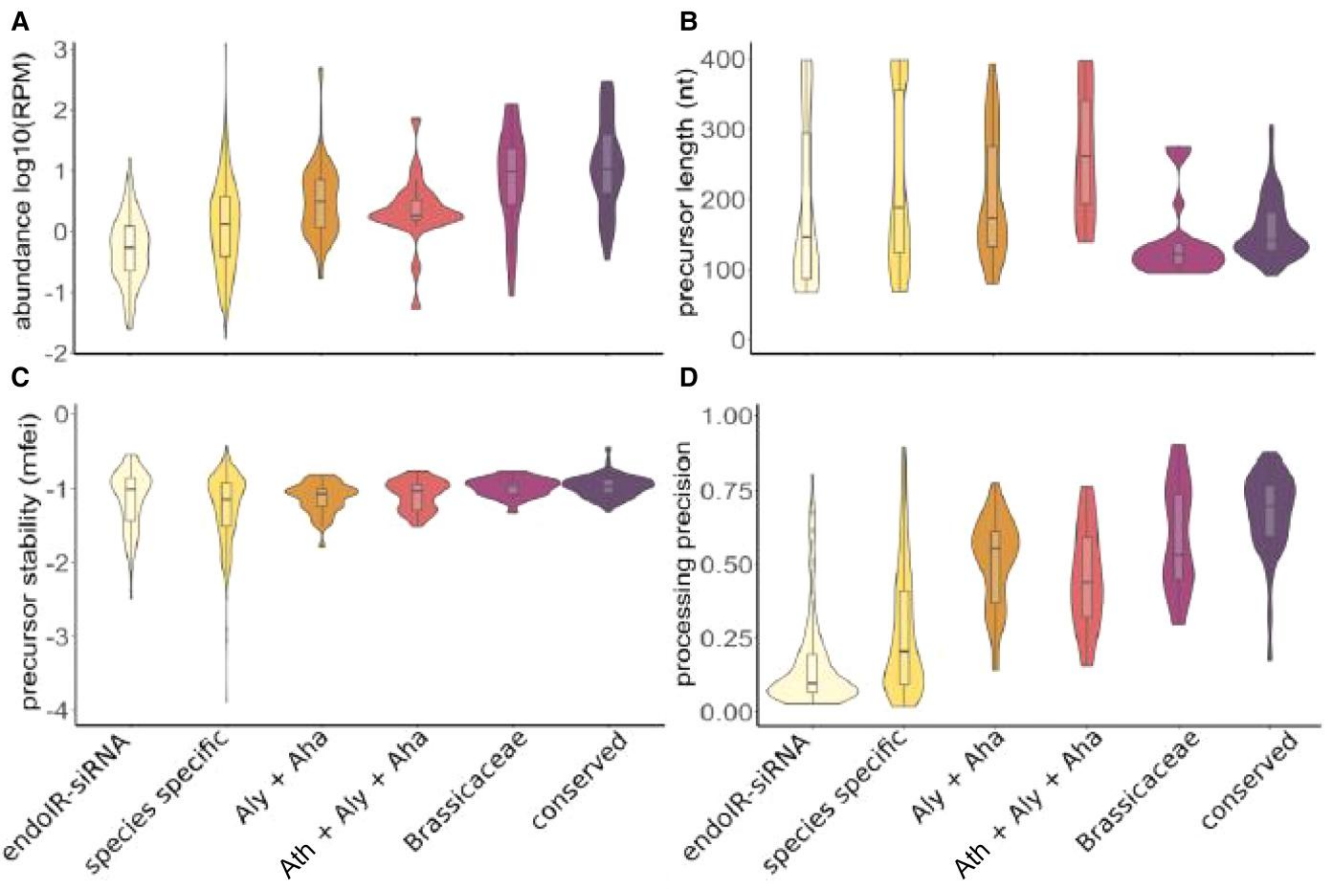
Graphical representation of miRNA evolution in *Arabidopsis lyrata* and *A. halleri*. **A)** Abundance of miRNAs, **B)** precursor length, **C)** precursor stability, and **D)** processing precision across endoIR-siRNA (24-nt sRNAs as negative control); species specific (*A.lyrata/Aly* or *A.halleri/Aha*); common miRNAs *Aly* and *Aha*; common miRNAs in A.thaliana/Ath, Aly and Aha; common miRNAs with Brassicaceae as distant plant family comparison; and conserved miRNAs. Figures adapted from Pavan et al. (2025) Fig. 5A and Fig. 3A–D.

Despite these insights, a major challenge remains in experimentally validating the biological function of these young, species-specific miRNAs. Nonetheless, Pavan et al. (2025) provide valuable insights into the transition of young miRNAs toward canonical status and highlight the dynamic nature of miRNA evolution in plants. Their study offers a framework that can be applied to other plant species to deepen our understanding of small RNA biology and evolutionary adaptation.

## Recent related articles in *The Plant Cell*

- Wang et al. (2024) reported on noncanonical long-loop precursors of miRNA858 in seed plants. This work challenges the current bioinformatics workflow that excludes long-loop precursors as viable candidates for miRNA biogenesis.
- Li et al. (2024) reviewed the role of biomolecular condensates, such as D-bodies for miRNA processing, in mediating RNA silencing in plants.
- Cao et al. (2025) showed that enhanced nuclear localization of HYPONASTIC LEAVES 1 (HYL1) is responsible for the upregulation of miRNA upon heat stress exposure rather than MIR gene upregulation.
- Park et al. (2023) demonstrated that nascent miRNA precursors are recognized by HYL1, allowing the recruitment of chromatin remodelers to MIRNA loci.

## Data Availability

No new data were generated or analysed in support of this article.

## Dive Curated Terms

The following phenotypic, genotypic, and functional terms are of significance to the work described in this paper:

AGO4 Gramene: AT2G27040
AGO4 Araport: AT2G27040

AGO1 Gramene: Os02g0672200
AGO1 Araport: Os02g0672200

HYL1 Gramene: AT1G09700
HYL1 Araport: AT1G09700

DCL1 Gramene: AT1G01040
DCL1 Araport: AT1G01040

## References

[koaf159-B1] Axtell MJ, Westholm JO, Lai EC. Vive la différence: biogenesis and evolution of microRNAs in plants and animals. Genome Biol. 2011:12(4):221. 10.1186/gb-2011-12-4-221 21554756 PMC3218855

[koaf159-B2] Cao Y, Zhang J, Zhao Z, Tang G, Yan J. Heat stress triggers enhanced nuclear localization of HYPONASTIC LEAVES 1 to regulate microRNA biogenesis and thermotolerance in plants. Plant Cell. 2025:37(6):koaf092. 10.1093/plcell/koaf092 40266261 PMC12142590

[koaf159-B3] Cuperus JT, Fahlgren N, Carrington JC. Evolution and functional diversification of MIRNA genes. Plant Cell. 2011:23(2):431–442. 10.1105/tpc.110.082784 21317375 PMC3077775

[koaf159-B4] Li Q, Liu Y, Zhang X. Biomolecular condensates in plant RNA silencing: insights into formation, function, and stress responses. Plant Cell. 2024:36(2):227–245. 10.1093/plcell/koad254 37772963 PMC10827315

[koaf159-B5] Lopez-Gomollon S, Baulcombe DC. Roles of RNA silencing in viral and non-viral plant immunity and in the crosstalk between disease resistance systems. Nat Rev Mol Cell Biol. 2022:23(10):645–662. 10.1038/s41580-022-00496-5 35710830

[koaf159-B6] Mencia R, Gonzalo L, Tossolini I, Manavella PA. Keeping up with the miRNAs: current paradigms of the biogenesis pathway. J Exp Bot. 2023:74(7):2213–2227. 10.1093/jxb/erac322 35959860

[koaf159-B7] Meyers BC, Axtell MJ. MicroRNAs in plants: key findings from the early years. Plant Cell. 2019:31(6):1206–1207. 10.1105/tpc.19.00310 31036598 PMC6588298

[koaf159-B8] Park J, Giudicatti AJ, Bader ZE, Han MK, Møller C, Arce AL, Xu Z-Y, Yang SW, Manavella PA, Yun D-J. The HIGH EXPRESSION OF OSMOTICALLY RESPONSIVE GENE15-HISTONE DEACETYLASE9 complex associates with HYPONASTIC LEAVES 1 to modulate microRNA expression in response to abscisic acid signaling. Plant Cell. 2023:35(8):2910–2928. 10.1093/plcell/koad132 37195876 PMC10396366

[koaf159-B9] Pavan F, Azevedo Favory J, Lacoste E, Beaumont C, Louis F, Blassiau C, Cruaud C, Labadie K, Gallina S, Genete M, et al The evolutionary history and functional specialization of microRNA genes in Arabidopsis halleri and A. lyrata. Plant Cell. 2025:koaf168. 10.1093/plcell/koaf168 40577601

[koaf159-B10] Ramos-Onsins SE, Stranger BE, Mitchell-Olds T, Aguadé M. Multilocus analysis of variation and speciation in the closely related species Arabidopsis halleri and A. lyrata. Genetics. 2004:166(1):373–388. 10.1534/genetics.166.1.373 15020431 PMC1470697

[koaf159-B11] Wang W-Q, Liu X-F, Zhu Y-J, Zhu J-Z, Liu C, Wang Z-Y, Shen X-X, Allan AC, Yin X-R. Identification of miRNA858 long-loop precursors in seed plants. Plant Cell. 2024:36(5):1637–1654. 10.1093/plcell/koad315 38114096 PMC11062470

