# Correction to: Emergence and evolution of canonical microRNAs: A case study in *Arabidopsis halleri* and *A. lyrata*

**DOI:** 10.1093/plcell/koag105

**Published:** 2026-05-29

This is a correction to: Ng, P.Q. Emergence and evolution of canonical microRNAs: A case study in *Arabidopsis halleri* and *A. lyrata*, *The Plant Cell*, Volume 37, Issue 7, July 2025, koaf159, https://doi.org/10.1093/plcell/koaf159

The original article included the following misleading or inaccurate statements:

1. A line in paragraph 5 of the article incorrectly stated that *A. thaliana* is the common ancestor of *A. halleri* and *A. lyrata*. Rather, *A. lyrata* and *A. halleri* are close living relatives of *A. thaliana. A. lyrata* and *A. thaliana* diverged from a common ancestor about 5 MYA, and *A. halleri* and *A. lyrata* diverged from each other about 1 MYA.
2. In paragraph 5, *A. halleri* is referred to as “*A. helleri”*. *A. halleri* is the correct spelling.
3. In the original Figure, a drawing of *A. lyrata* and *A. halleri* to the left of the lettered panels included a timeline with an arrow depicting 1 million years, giving the impression that one descended from the other, which is not the case, as described above.
4. In the figure legend, “edoIR-siRNA” should be “endoIR-siRNA.”

The Figure has been revised to omit the drawings and timeline. The lettered panels in the figure, reproduced from Figure 3A-3D of Pavan et al. (2025), remain unchanged. The figure shows that, during *Arabidopsis* miRNA evolution, there was a tendency for a decline in miRNA gene expression (panel A) and average hairpin (miRNA precursor) length (panel B), and an increase in the stability of the hairpin structure (panel C) and average processing precision (panel D).

Original Figure:

**Figure:**
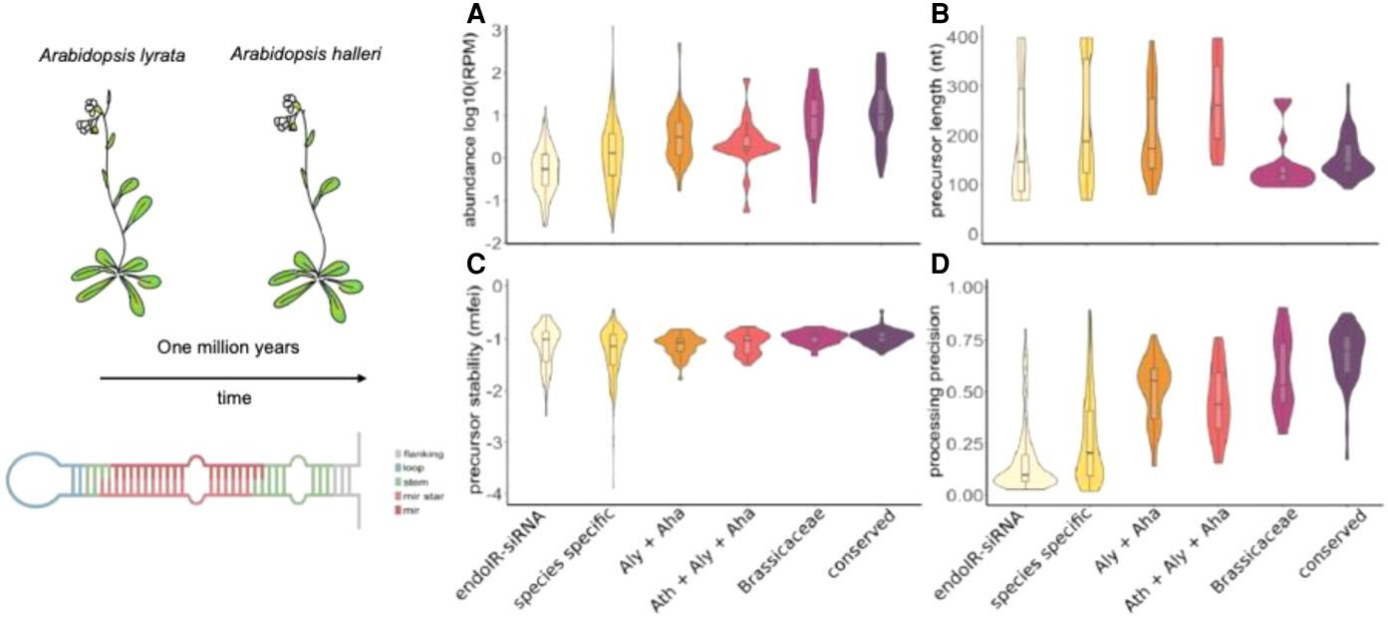
Original Figure legend: **Figure.** Graphical representation of miRNA evolution in *Arabidopsis lyrata* and *A. halleri*. A) Abundance of miRNAs, B) precursor lengths, C) precursor stability, and D) processing precision across edoIR-siRNA (24nt sRNAs as negative control), species specific (*A.lyrata/Aly* or *A.halleri/Aha*), common miRNAs *Aly* and *Aha*, common miRNAs in *A.thaliana/Ath*, *Aly* and *Aha*, common miRNAs with Brassicaceae as distant plant family comparison and conserved miRNAs. Figures adapted from Pavan et al. (2024) Fig. 5A and Fig. 3A-D.

Revised Figure:

**Figure:**
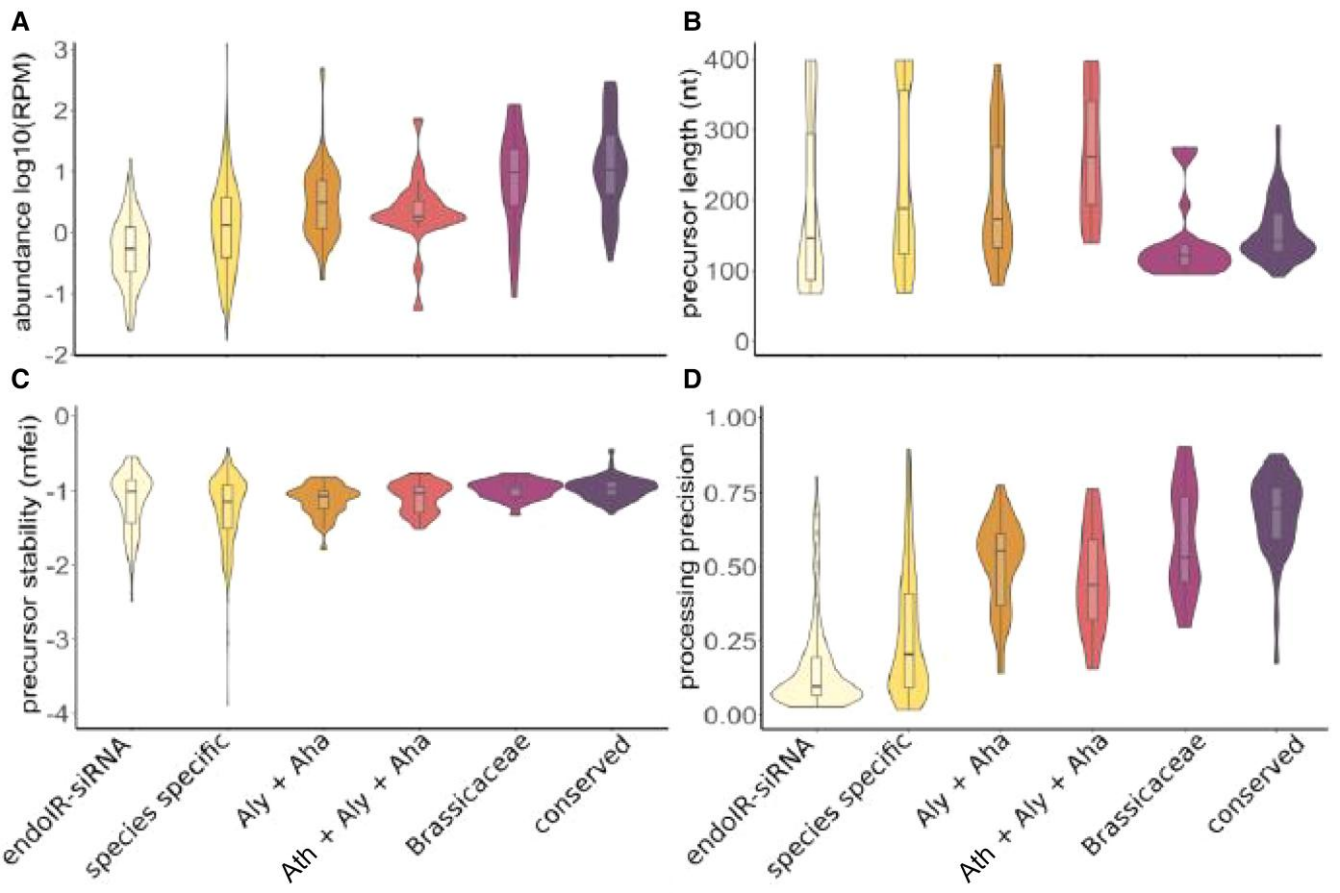


Revised Figure legend:

**Revised Figure.** Graphical representation of miRNA evolution in *Arabidopsis lyrata* and *A. halleri*. **A)** Abundance of miRNAs, **B)** precursor length, **C)** precursor stability, and **D)** processing precision across endoIR-siRNA (24-nt sRNAs as negative control); species specific (*A.lyrata/Aly* or *A.halleri/Aha*); common miRNAs *Aly* and *Aha*; common miRNAs in *A.thaliana/Ath*, *Aly* and *Aha*; common miRNAs with Brassicaceae as distant plant family comparison; and conserved miRNAs. Figures adapted from Pavan et al. (2025) Fig. 3A–D.

